# An asynchronous artifact-enhanced electroencephalogram based control paradigm assisted by slight facial expression

**DOI:** 10.3389/fnins.2022.892794

**Published:** 2022-08-16

**Authors:** Zhufeng Lu, Xiaodong Zhang, Hanzhe Li, Teng Zhang, Linxia Gu, Qing Tao

**Affiliations:** ^1^School of Mechanical Engineering, Xi’an Jiaotong University, Xi’an, China; ^2^Shaanxi Key Laboratory of Intelligent Robot, Xi’an Jiaotong University, Xi’an, China; ^3^Department of Biomedical and Chemical Engineering and Sciences, College of Engineering and Science, Florida Institute of Technology, Melbourne, FL, United States; ^4^School of Mechanical Engineering, Xinjiang University, Wulumuqi, China

**Keywords:** asynchronous, EEG, EEG-based control, artifacts, facial-expression

## Abstract

In this study, an asynchronous artifact-enhanced electroencephalogram (EEG)-based control paradigm assisted by slight-facial expressions (sFE-paradigm) was developed. The brain connectivity analysis was conducted to reveal the dynamic directional interactions among brain regions under sFE-paradigm. The component analysis was applied to estimate the dominant components of sFE-EEG and guide the signal processing. Enhanced by the artifact within the detected electroencephalogram (EEG), the sFE-paradigm focused on the mainstream defect as the insufficiency of real-time capability, asynchronous logic, and robustness. The core algorithm contained four steps, including “*obvious non-sFE-EEGs exclusion*,” “*interface ‘ON’ detection,”* “*sFE-EEGs real-time decoding*,” and “*validity judgment.”* It provided the asynchronous function, decoded eight instructions from the latest 100 ms signal, and greatly reduced the frequent misoperation. In the offline assessment, the sFE-paradigm achieved 96.46% ± 1.07 accuracy for *interface* “*ON” detection* and 92.68% ± 1.21 for *sFE-EEGs real-time decoding*, with the theoretical output timespan less than 200 ms. This sFE-paradigm was applied to two online manipulations for evaluating stability and agility. In “*object-moving with a robotic arm*,” the averaged intersection-over-union was 60.03 ± 11.53%. In “*water-pouring with a prosthetic hand*,” the average water volume was 202.5 ± 7.0 ml. During online, the sFE-paradigm performed no significant difference (*P* = 0.6521 and *P* = 0.7931) with commercial control methods (i.e., FlexPendant and Joystick), indicating a similar level of controllability and agility. This study demonstrated the capability of sFE-paradigm, enabling a novel solution to the non-invasive EEG-based control in real-world challenges.

## Introduction

To regulate human–machine interaction in a natural way, electroencephalogram (EEG)-based control has been considered as a promising form. Non-invasive EEGs with lower cost and free surgery risk have higher universal application potentials (Casson et al., 2010). To establish the direct pathway between the brain and the peripherals, several paradigms have been developed to arouse typical responses of the brain activity (Abiri et al., 2019). The widely used paradigms include motor imagery (MI; Jin et al., 2019), slow cortical potential (SCP), P300, steady-state visual evoked potential (SSVEP; Jin et al., 2021a) and so on (Hwang et al., 2013). Over the past decades, research on the brain control interface (BCI) paradigms have achieved significant progress, such as P300 and SSVEP in high-speed screen spellers (Hoffmann et al., 2008; Chen et al., 2015), SCP in thought translation device (Birbaumer et al., 1999), and MI in the motor recovery after stroke (Sharma et al., 2006). Progressively, the BCI focuses not only on restoring communication and control in severely paralyzed patients, but also proves its usage for healthy people (Nijholt and Tan, 2008).

In the aspect of EEG-based robotic control or electromechanical system manipulation, several impressive works, such as, SSVEP-based control of wheelchair (Muller et al., 2011), robotic arm (Chen X. G. et al., 2018) and quadcopter (Wang et al., 2018), MI-based operation of wheelchair navigation (Carlson and Millan, 2013), robotic arm in reach, and grasp tasks (Meng et al., 2016; Edelman et al., 2019), the shared control of robotic grasping (Chen et al., 2019), and the quadcopter control in three-dimensional space (LaFleur et al., 2013), P300-based robotic guide (Chella et al., 2009), wheelchair-mounted robotic ann system (Palankar et al., 2009), have been proposed world-wide by researchers. Besides the BCIs, different hybrid BCIs (hBCIs) are also developed to enrich the function, including hBCI with SSVEP and EOG in controlling the robotic arm (Zhu et al., 2020), SSVEP, and MI in orthosis operation (Pfurtscheller et al., 2010), MI and error related potential for position control (Bhattacharyya et al., 2017), combination of MI, SSVEP, and eye blink in quadcopter flight control (Duan et al., 2019), P300 and SSVEP in the application to wheelchair driving (Li et al., 2013) and the ideogram and phonogram writing with robotic arm (Han et al., 2020), MI and P300 in speed and direction controlling (Long et al., 2012), SSVEP-MI-EMG-hBCI for robotic arm in writing tasks (Gao et al., 2017), and so on. As above, the mainstream paradigms adopted in the neuro-based electromechanical system control include SSVEP, MI, P300, and their combination and hybrid.

Considering more general cases and scenarios, the visual-stimulated paradigm (i.e., SSVEP, P300) has its limitation for an additional visual stimulator and period for visual evocation, and the MI requires extra adaptation to paradigm-self and high concentration. By viewing the neuro-based control as a promising control method, it needs to satisfy the basic requirements of real-time, precision, user-friendliness, and easiness, similar to the traditional control approaches. Focusing on the limitation of mainstream paradigms in electromechanical system manipulation, in 2018, a BCI assisted by facial expression (FE-BCI) had been proposed by our research group (Zhang et al., 2016; Li R. et al., 2018; Lu Z. et al., 2018), which has the characteristics of no additional user adaption, no extra stimulators, and no nerve adaptability (Li R. et al., 2018). From the aspect of neurophysiology, the mechanism of facial expression had been studied (Li R. et al., 2018) and the functional connectivity analysis was conducted (Lu Z. F. et al., 2018), which revealed the separability of facial-expression assisted brain signals and guided the signal processing. When performing facial expressions, our previous study proved that both the EEG component and the electromyogram (EMG) component can be detected by the EEG electrodes at the same time, and each component can be decoded to provide the instruction for external device operation (Li R. et al., 2018; Zhang et al., 2020). Benefiting from the coexistence of EEG and EMG components, enhanced by the EMG artifacts within EEG band, without separating these components, it enabled the capability for real-time decoding and control (each output generated from the latest 100 ms inputs) (Lu Z. F. et al., 2018), and realized the semi-asynchronous logic (Lu et al., 2020).

In this study, as an update, an asynchronous artifact-enhanced EEG-based control paradigm assisted by slight facial-expression (sFE-paradigm) is proposed to improve its practical performance for comprehensive and complex daily situations. Both offline and online experiments were conducted. The effective connectivity analysis of sFE-paradigm was demonstrated to delineate the interaction among EEGs. The methodology of sFE-paradigm, including computation logic, asynchronous strategy, and detailed steps, was illustrated. The offline performance and online controllability were assessed. The novel aspects of this new paradigm are as follows: (1) An integral asynchronous strategy is developed to enable users to switch on/off the paradigm in their will; (2) The sFE provides the possibility to distinguish the exaggerated facial expressions in daily communication during EEG-based-control, and also increases the aesthetics, which ensures the suitability for patients who can hardly complete exaggerated facial expressions; (3) The instruction sets are expanded with the support of an improved core algorithm, combining the deep-learning framework; and (4) A validity judgment step is added to decrease the frequent misoperations exposed in the traditional literal translation mode.

## Materials

### Electroencephalogram recording

**FIGURE 1:**
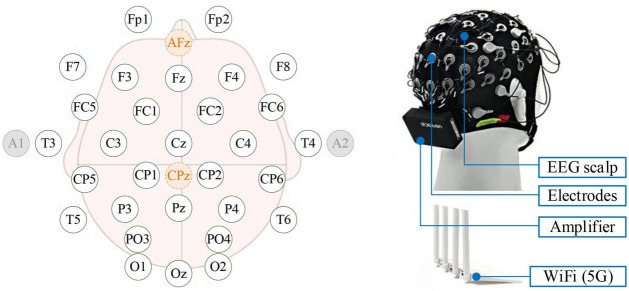
The Neuracle device and its electrode placement. By system default, the Afz is the ground, CPz is the reference, and A1 and A2 are EOGs.

The commercial EEG acquisition system (Neuracle Technology Co., Ltd.) has 34 electrodes (including 30 EEG channels, 1 reference, 1 ground, and 2 EOG channels) with 1,000 Hz sampling rate and WiFi module (Figure 1). The reference was placed at CPz, and the ground was placed at AFz (Yao, 2001). During the experiment, impedances were kept below 5 kΩ.

### Subjects

Sixteen healthy subjects (25–38 years of age, 13 men and 3 women) participated in the experiments, without any known cognitive deficits and prior experience (World Medical Association, 2013). These sixteen subjects were divided into two groups, where six subjects (S1-S6) participated only in the offline experiment for offline algorithm assessment and software development, while the other ten subjects (S7–S16) participated directly in the online experiment to verify the practicability of the sFE-paradigm. Before the experiment, an instruction video was displayed to subjects to illustrate the experimental setup and the difference between the sFE and facial expression in regular amplitude.

### Offline experiment

**FIGURE 2:**
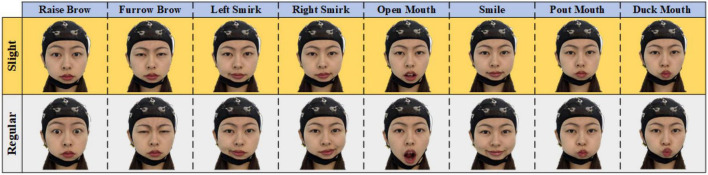
Eight slight-facial expressions (sFEs) adopted in this research and their comparison with the regular amplitude.

To develop an asynchronous sFE-paradigm, EEGs under resting-state, aroused by sFE and under relaxing-state were collected. A total of eight easy-to-execute sFEs were adopted. EEGs with the same facial expressions but in regular amplitude were also collected for comparison. Figure 2 illustrates eight sFEs and their comparison with the regular amplitude.

#### Section I: Slight facial expression

**FIGURE 3:**
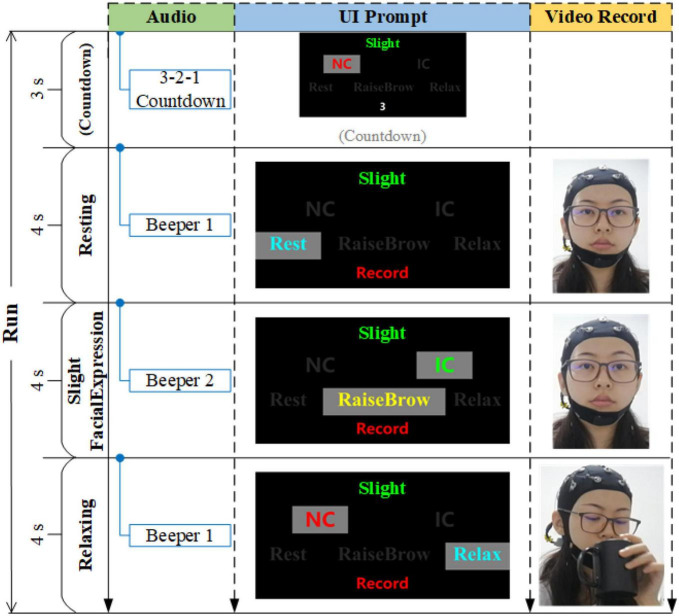
The timing diagram of one trial, where IC represents “in-control,” and NC represents “non-control”.

Eight sFEs were performed by each subject following the on-screen prompts. Each sFE was conducted for five sessions consisted of eight trials. In each trial, 3 s countdown, 4 s resting-state, 4 s sFE-state, and 4 s relaxing-state were contained in turn (Figure 3). At the resting-state, the subjects were asked to avoid extra movements. At the sFE-state, the subjects followed the on-screen prompts to make the designated sFE and hold it for 4 s. At the relaxing-state, the subjects were free for any volunteered movement, including but not limited to talking, drinking, reading, writing, physical activity, and so on.

#### Section II: Facial expression (regular amplitude)

In the regular amplitude section, the same session settings as the sFE phase were adopted. For comparison, each facial expression was conducted for only 2 trials.

#### Section III: Relaxing-state

Three additional trials of EEGs under relaxing were collected, including one trial for reading and talking, one trial for drinking and eating, and one trial for physical moving. Each trial lasted for 96 s.

### Online experiment

To verify the feasibility and the practicality of an sFE-paradigm for peripheral application, subjects S7–S16 participated in the online experiment without prior experience with the offline experiment. Two specific external device manipulation tasks, atypical versus daily, focused on stability and agility, respectively, were conducted. In both tasks, EEGs were sent wirelessly by Wi-Fi to the computer (Windows 10, i5-6500 CPU, 3.20GHz, GTX 960 GPU). The stepping control was adopted by both the devices. During the online experiment, an sFE-paradigm software developed after the offline assessment was used, operated manually by the subjects themselves. Following the graphical user interface (GUI), subjects first login to the software, and then completed the training data collection work. One’s individual parameters and the classifiers were automatically calculated by the software. Finally, the online manipulation tasks were started by pressing the “Start”’ button on the GUI by subjects themselves. Compared with the offline, the training data acquisition process in the online is consistent with the offline setup, but with fewer trials.

#### Task one: Object-moving with a robotic arm

**FIGURE 4:**
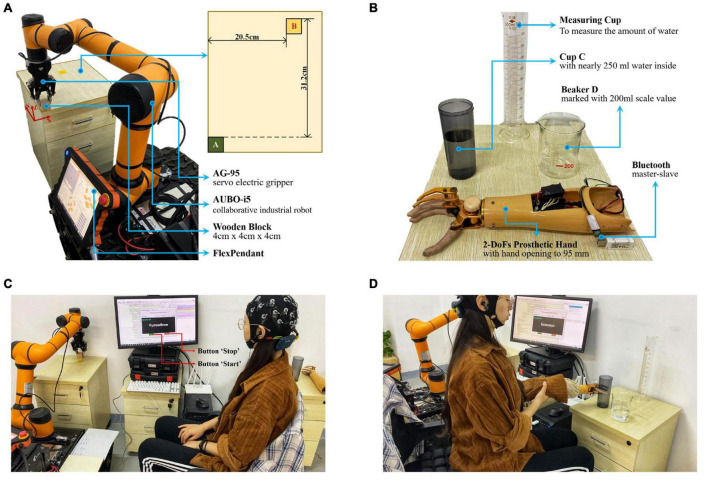
The illustration for online experiment. **(A)** The initial state of the assembled robotic arm system in *Task one* (object-moving), and the plan view of the position A and B. **(B)** Experiment scene layout and the 2-DoFs prosthetic hand of *Task two* (water-pouring). **(C)** Experimental scene graph for *Task one* (object-moving). **(D)** Experimental scene graph for *Task two* (water-pouring).

In this task, subjects were required to use this proposed sFE-paradigm to first switch on the system, then operate the robotic arm to move a wooden block (4 cm × 4 cm × 4 cm) from its initial position A to the position B (Figure 4A), and then switch off the system. The robotic arm used was the assembly of an AUBO-i5 collaborative industrial robot (six DoFs, provided by AUBO Robotics China Co., Ltd.) equipped with an AG-95 servo-electric gripper (provided by DH-Robotics Technology Co. Ltd.). Before each trial, the robotic arm restored its initial state, with the block grabbed. To move the block, subjects need to first lift it, then adjust its position, and finally lower it and open the electric gripper to ensure a stable placement. Table 1 shows the instruction list. The experimental scene is graphed in Figure 4C. Before starting, subjects first had 5 min to get familiar with the operation for sFE-paradigm-based robotic arm; then this task was repeated for five trials. In each trial, subjects began to complete the task after pressing the “Start” button on the GUI by themselves. If the task was not finished within 5 mins or if the block had fallen halfway, this trial would be deemed as a failure. The deviation for the same task using FlexPendant was recorded as a reference.

**TABLE 1:** The instruction list of AUBO-i5 system.

Instruction	System Action	Axis	Stepping
“Right”	Horizontally move right	+X	30 mm
“Left”	Horizontally move left	–X	2 mm
“Forward”	Horizontally move forward	+Y	20 mm
“Backward”	Horizontally move backward	–Y	3 mm
“Up”	Vertically rise up	+Z	30 mm
“Down”	Vertically lower down	–Z	$\left\{ \begin{array}{l} 10\text{mm}(\text{Z}>21\text{mm})\\ 3\text{mm}(\text{Z}\leq 21\text{mm}) \end{array}\right.$
“Open”	Electric gripper open	–	–

#### Task two: Water-pouring with a prosthetic hand

In this task, the subjects were required to use the sFE-paradigm to first switch on the system, then operate the prosthetic hand to pour 200 ml water from cup C to beaker D, and then switch off the system (Figure 4B). The prosthetic hand used was customized by Danyang Artificial Limb Co., Ltd. with 2-DoFs (wrist and finger joints) and Bluetooth module. Before each trial, the prosthetic hand was kept in front of cup C with the hand opened to the maximum (95 mm), and the water initially stored in the cup C was nearly 250 ml. To pour the water, the subjects need to grasp cup C first, then lift and whirl it to transfer demanded amount of water, and finally slightly lower it and open the palm. Table 2 lists the instruction of the 2-DoFs prosthetic hand. Figure 4D shows the experimental scene. Similarly, subjects had 5 min first to get familiar with the system, then this task was repeated for five trials. Each trial started with the ‘Start’ button being pressed by the subject-self and would be viewed as a failure if not completed within 3 min or the cup was dropped halfway. The deviation to perform water-pouring with this prosthetic hand but controlled by Joystick was also recorded as a reference.

**TABLE 2:** The instruction list of the prosthetic hand.

Instruction	System action	Stepping
“Open”	Palm open	7.0 mm
“Close”	Palm close	10.5 mm
“Extorsion”	Wrist external rotation	11.0°
“Intorsion”	Wrist internal rotation	12.0°

### Method

#### Brain connectivity analysis

Brain connectivity assesses the integration of cerebral areas by identifying variations in activation within and interactions between brain areas (Friston, 2011; Sakkalis, 2011; Li Y. Q. et al., 2018). As one major aspect, effective connectivity is defined as the direct or indirect influence that one neural system exerts over another (Horwitz, 2003). To obtain a better insight into this sFE-paradigm, orthogonalized partial directed coherence (OPDC) was used to measure the dynamic directional interactions among brain regions under sFEs.

Orthogonalized partial directed coherence, as an orthogonalized version of the classical partial directed coherence (PDC; Sommerlade et al., 2009), focuses on reducing the spurious co-variability resulted from the similarity in several EEGs affected by volume conduction (Nolte et al., 2004), which may be falsely perceived as connectivity (Gomezherrero, 2010). The OPDC developed out of the concept of Granger-causality (Granger, 1969), investigating the information flow within coupled dynamical networks based on multivariate autoregressive (MVAR) models (Geweke, 1982), detecting not only direct but also indirect pathways linking interacting brain regions (Baccala and Sameshima, 2001). Based on the dual extended Kalman filter-based time-varying PDC analysis (Omidvarnia et al., 2011), by orthogonalizing the imaginary part of the coherence (Nolte et al., 2004), the OPDC mitigates the common result of volume conduction effects. The estimating process of OPDC is as follows (Omidvarnia et al., 2014):

##### Multivariate autoregressive model

For time series *y*(*n*) ∈ *Rc*^ch^ with *L* samples (where *n* = 1,…,*L*), the MVAR model is defined as (Hytti et al., 2006):


(1) $$\left[\begin{array}{c} y_{1}\,\left(n\right)\\ \vdots \\ y_{c\,h}\,\left(n\right) \end{array}\right]=\sum_{r=1}^{m}\left[\begin{array}{ccc} a_{1,1}^{r} & \cdots & a_{1,c\,h}^{r}\\ \vdots & \ddots & \vdots \\ a_{c\,h,1}^{r} & \cdots & a_{c\,h,c\,h}^{r} \end{array}\right]\,\left[\begin{array}{c} y_{1}\,\left(n-r\right)\\ \vdots \\ y_{c\,h}\,\left(n-r\right) \end{array}\right]+\left[\begin{array}{c} w_{1}\,\left(n\right)\\ \vdots \\ w_{c\,h}\,\left(n\right) \end{array}\right]$$

where ${\left[y_{1}(n)\cdots y_{ch}(n)\right]}^{T}$ is the current value of each time series, *ch* denotes the number of channels, the real-valued parameter $a_{p,q}^{r}\left(p=1,\ldots ,ch;q=1,\ldots ,ch\right)$ in matrices *A*_*r*_ represents the predictor coefficient between the current value of channel *p* and the past information of channel *q* at the delay *r*, *m* is the model order indicating the number of previous data points used for modeling, and ${\left[w_{1}(n)\cdots w_{ch}(n)\right]}^{T}=w(n)$ is a normally distributed real-valued zero-mean white noise vector representing one-step prediction error. The instantaneous effect among channels is explained by correlations among the off-diagonal element within the covariance of *w*(*n*) (Faes and Nollo, 2010). The optimum order *m* was estimated using Akaike information criterion (AIC) (Akaike, 1971).

##### Time-varying generalized orthogonalized partial directed coherence

To detect the coherence between channels in discrete frequency, *A*_*r*_ in the time-varying MVAR model is transformed into frequency domain:


(2) $$A\,\left(n,f\right)=I-{\sum_{r=1}^{m}A_{r}\,\left(n\right)\,z^{-r}|}_{z=e^{j\,2\,\pi \,f}}$$

where *I* is the identity matrix and the frequency *f* varies from 0 to the Nyquist rate. To alleviate the effect of mutual sources within surface EEGs affected by spatial smearing in the tissue layer, the orthogonalization process is done at the level of MVAR coefficients (Omidvarnia et al., 2012). The generalized version of OPDC by taking the effect of time series scaling into consideration is:


(3) $$\overset{∼}{\underset{p,q}{\Psi }}\left(n,f\right)=\frac{1}{\lambda_{p,p}^{2}}\frac{\left|\mathrm{Real}\left\{{\text{A}}_{\text{p,q}}\left(\text{n,f}\right)\right\}\right|}{\sqrt{a_{q}^{H}\left(n,f\right)\Sigma_{w}^{-1}a_{q}\left(n,f\right)}}\frac{\left|\mathrm{Imag}\left\{{\text{A}}_{\text{p,q}}\left(\text{n,f}\right)\right\}\right|}{\sqrt{a_{q}^{H}\left(n,f\right)\Sigma_{w}^{-1}a_{q}\left(n,f\right)}}\text{if}p\neq q$$

where $\overset{∼}{\underset{p,q}{\Psi }}$ denotes the gOPDC from channel *q* to channel *p*, λ_*p*,*p*_ are the diagonal elements of covariance matrix $\sum_{w}=\langle ww^{T}\rangle$ (where ⟨⋅⟩ is the expected value operator), the superscript *H* denotes the Hermitian operator and *a*_*q*_(*n*,*f*) is the *q*th column of *A*(*n*,*f*). For detailed mathematical derivation of gOPDC, refer to Omidvarnia et al. (2014).

### Component analysis of slight-facial expressions-electroencephalogram

To better understand the sFE-EEG and form guidance on the signal processing, the component analysis was conducted to illustrate the dominate component in the sFE-EEG. To separate the components in sFE-EEGs, both the fast independent component analysis (FastICA) (Hyvarinen and Oja, 2000) and the noise-assisted multivariate empirical mode decomposition (NA-MEMD) (Chen X. et al., 2018) were taken, of which FastICA focused on multi-channels combined and NA-MEMD focused on every single channel.

After the components were separated, sample entropy (SampEn) as one effective way to identify the complexity of changes in biological signals was selected as the indicator (Cuesta-Frau et al., 2017). Under the same circumstance, the randomness of the EMG component shall be much stronger than that of the EEG component, leading to a much larger entropy. Therefore, when exceeding the threshold of sample entropy, it means that the component contains a lot of EMG information. The SampEn can be calculated as follows:


(4) $$\text{SampEn}\,\left(m,r,N\right)=-\ln\left[\frac{A^{m}\,\left(r\right)}{B^{m}\,\left(r\right)}\right]$$

where *m* is a constant, *r* is the tolerance, *N* is the sequence length, *B^m^*(*r*) is the probability of matching *m* points with tolerance *r*, and *A^m^*(*r*) is the probability of matching *m* + 1 points. In this study, *m* = 2, *r* = 0.2*std, where *std* represents the standard deviation. Referring to the previous literature and experimental experiences (Friesen et al., 1990; Liu et al., 2017), the specific threshold of SampEn was set as 0.45. When exceeding the threshold, the corresponding component would be identified as containing more EMG information than EEG.

### Asynchronous interface of slight-facial expressions-paradigm

To meet the goal of practicality, the fundamental demands for one control approach are stability, accuracy, real-time, and user-friendliness. As for an EEG-based control paradigm, the specific technical details can be listed as follows: (1) Using fewer EEGs for each decoding, to improve the real-time ability; (2) Providing asynchronous strategy, to ensure users start or stop paradigm as needed; (3) Adopting a paradigm which requires no additional stimulators and adaptation, and has no strict requirement for high concentration; (4) Ensuring high decoding accuracy with adequate instruction set; (5) Giving an allowance to users’ unrelated movements, without affecting the manipulation performance; (6) Reducing the common problem of misoperation, thus enhancing the stability. In the real-time aspect, Lauer et al. (2000) stated that any delay greater than 200 ms would degrade the performance of one neuro-based task accomplishment. Evidenced by our previous study, the separation of EMG component would largely increase the system process time (Li R. et al., 2018), and the coexistence of EMG artifact within EEG bands makes large contribution to compress the decoding time and improve accuracy (Lu Z. F. et al., 2018). Thus, to improve the real-time ability, in this sFE-paradigm, artifact would be used as the enhancement for system performance without separating it independently. Further, considering the process of data acquisition, signal processing, and instruction generation, the window length of EEGs used for once decoding in sFE-paradigm is chosen as 100 ms.

**FIGURE 5:**
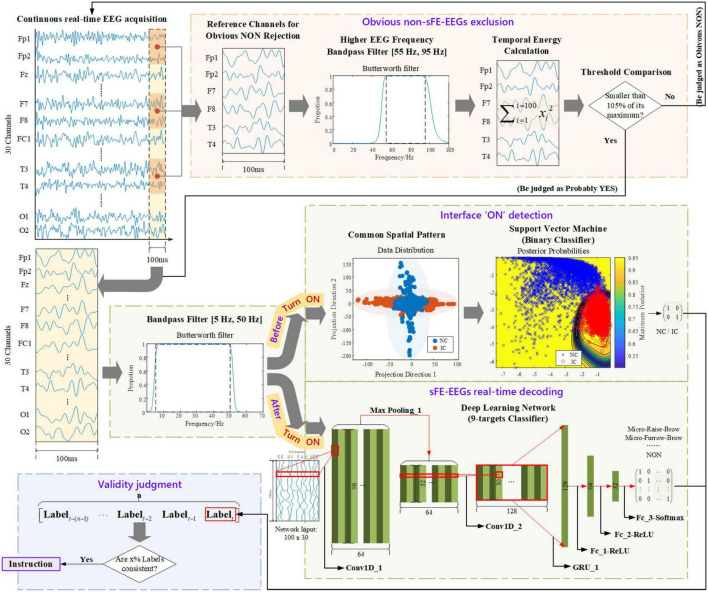
The detailed algorithm of slight-facial expression-paradigm.

The processing algorithm in sFE-paradigm is demonstrated in Figure 5. The algorithm included four parts: (1) ‘Is this probably sFE-EEGs or obvious “NON,” aiming at excluding obvious non-sFE-EEGs at the beginning? (2) “Asynchronous interface ON detection,” “turning on or off the paradigm depends on users” will; (3) “sFE-EEGs real-time decoding,” classifies the sFE-EEGs and keeps an extra “NON” to give a respite for users (meanwhile a hold-on command for the system); (4) “Validity judgment,” judging the validity of the label first to reduce misoperation instead of directly converting it into instruction. Such a step-by-step design is used to shorten the processing time for inputs unrelated to current system status (e.g., exaggerated facial expression EEGs would be rejected from the very beginning; EEGs would not be complexly classified until the asynchronous interface has been turned ON) meanwhile offering an allowance for other slightly physical movements, and improving the stability.

#### Step A: Obvious non-slight-facial expressions-electroencephalogram exclusion

The first step aims at using a simple computation to exclude inputs that are apparently non-sFE-EEGs, while not let go of any probably sFE-EEGs. To save the computing time and to improving the efficiency, Threshold Comparison, a junior method was chosen:.

**FIGURE 6:**
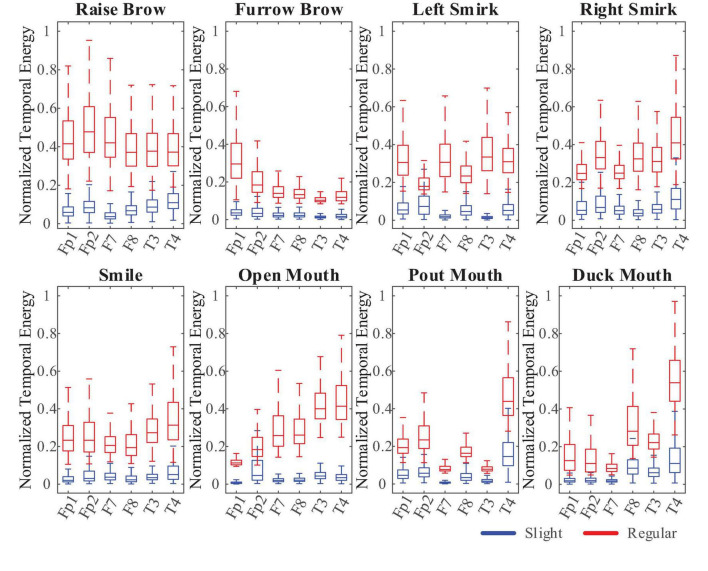
The comparison boxplot of normalized temporal energy.

Due to the EMG artifact, when performing regular or exaggerated facial expressions, channels placed closer to facial muscles (Fp1, Fp2, F7, F8, T3, and T4) tend to show wider amplitude range than sFEs, leading to larger temporal energy in higher EEG frequency (55 Hz, 95 Hz), as shown in Figure 6. These six channels were used as reference channels for obvious “NON” exclusion. Instead of being uniformly larger than one constant, the temporal energies are relatively larger only when compared to each reference channel itself. Thus, a separate threshold was set for each reference channel, as the 105% of one’s highest temporal energy among eight sFEs. The Threshold Comparison was designed as: When facing the latest unknown 100 ms inputs, if there exists one or more channels whose temporal energy exceeds its own threshold, the current EEGs would be judged as “NON” and be excluded. Those mistakenly accepted EEGs, similar to but not sFE-EEGs, would be processed by the next step.

#### Step B: Interface “ON” detection

This step enables the users to start the sFE-paradigm whenever they need, which means the system would not reply to any EEGs and keep a standby mode until the interface has been turned on. Such design guarantees the users the option of not controlling the peripherals and handling other affairs with the EEG cap worn and all devices powered on. To switch between the non-control (NC) state and the in-control (IC) state, one of the eight sFEs was chosen as the state-switching sFE: slight-raise-brow (sRB). The decoding for asynchronous “ON” is realized by a binary classifier. Only sRB-EEGs were labeled as “ON,” while other EEGs including resting, relaxing, other sFEs, and regular or exaggerated facial expressions, and EEGs mistakenly accepted by *Step A* were all labeled as “else”. Instead of deep learning algorithms, traditional machine learning algorithm support vector machine (SVM) was adopted to speed up the process.

Considering the low frequency solution resulting from the short time window, feature engineering was realized by a classic spatial filtering method: common spatial pattern (CSP) (Lu Z. F. et al., 2018; Zhang et al., 2019; Wang et al., 2020; Jin et al., 2021b). The CSP features were calculated as:


(5) $$f_{k}=\frac{\log\left(v\,a\,r\,\left(Y_{i,k}^{j}\right)\right)}{\sum_{k=1}^{2\,m}\log\left(v\,a\,r\,\left(Y_{i,k}^{j}\right)\right)},k=1,\ldots ,2\,m$$

where ${\text{Y}}_{i}^{j},i\in \left\{1,2\right\}$ is the new signal generated through the spatial filters from raw data, in which *i* indicates the *i^th^* category and *j* indicates the *j^th^* sample, *k* is the number of pairs of spatial filters which was set to be 2 in this study.

Corresponding to pair number, a 4-dimensional feature vector was formed. The kernel function in SVM was set to be scaled Gaussian (Eq. 6), according to the F1 score (Derczynski, 2016). Refer to Results for a detailed comparison.


(6) $$\left\{ \begin{array}{c} \text{G}\,\left(x_{j},x_{k}\right)=\exp\left(-\gamma \,{||x_{j}-x_{k}||}^{2}\right)\\ \gamma =\frac{1}{n_{f\,e\,a\,t\,u\,r\,e}*v\,a\,r\,\left(X\right)} \end{array}\right.$$

where G(*x*_*j*_,*x*_*k*_) denotes elements in the Gram matrix, γ is the hyperparameter for Gaussian kernel, *X* is the features sets and *n*_*feature*_ is the dimension of feature channels. Every 100 ms EEGs that passed through *Step A* was re-filtered into the typical EEG band as (5 Hz, 50 Hz) with all 30 channels and processed by this *Step B*.

#### Step C: Slight-facial expressions-electroencephalograms real-time decoding

After switching ON the sFE-paradigm, the algorithm enters the second stage: *sFE-paradigm based real-time control*. In the second stage, similarly, only those latest 100 ms EEGs accepted by *Step A* can enter *Step C*. In this step, nine targets were set in total, including seven sFEs corresponding to specified control instructions, the state-switching sFE corresponding to interface OFF, and a “NON” category as the hold-on command for system. Instead of setting a separate asynchronous “OFF” detection ahead, such a design with the control instruction and interface OFF combined together, is to speed up the efficiency. Otherwise, any sFE-EEGs intend for control have to pass through two classifiers (Lu et al., 2020).

This multi-classification was realized by the deep-learning framework. To take full advantage of the translation invariance and the spatial hierarchy of the convolutional neural network (CNN; Bengio and Lecun, 1997), meanwhile retaining the time sequential characteristics of EEGs, the variant of recurrent neural network (RNN; Rumelhart et al., 1986) and the CNN were combined. The classifier structure (namely sFE-Net) was summarized in Table 3. The input of sFE-Net was 30-channel EEGs with 100 sample points, which are judged by *Step A* ahead as “probably yes” and re-filtered into (5 Hz, 50 Hz). The outputs of sFE-Net were nine labels with one-hot code, which correspond to eight sFEs and a “NON”.

**TABLE 3:** Summary of sFE-Net.

Layer (type)	Method	Parameter	Value
Conv1D_1	Temporal dimension	Kernel dize	3
		Strides	1
		Filters	64
Pooling1D_1	Max pooling	Pool dize	3
		Strides	3
		Padding	Valid
Conv1D_2	Temporal dimension	Kernel size	3
		Strides	1
		Filters	128
GRU_1	Time domain	Activation	tanh
		Recurrent activation	Sigmoid
		Dropout	0.1
		Recurrent dropout	0.1
		Return sequences	False
		Units	128
Dense	–	Units	64
		Activation	ReLU
Dense	–	Units	32
		Activation	ReLU
Dense	One hot	Units	9
		Activation	Softmax
Loss function	Categorical cross entropy	–	–
Optimizer	RMSprop	Learning rate	0.001
Training acceleration	Batch	Batch size	128

#### Step D: Validity judgment

For realistic manipulation tasks, any misoperation shall result in a huge impact on the execution. To improve the robustness, instead of directly translating each decoded label into instruction, a validity judgment was added. Labels generated from *Step B* and *Step C* shall pass through *Step D*. Such a judgment was simply realized by the consistency checking: Among *n* decoded labels, if *x*% of them are consistent with the current *n*th, then the *n*th will be regarded as valid and translated into an instruction.

**FIGURE 7:**
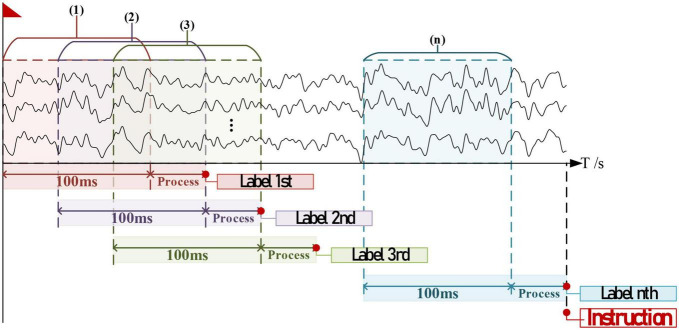
The timing diagram for one valid instruction generation, supposed that all collected data were slight-facial expression (sFE)- electroencephalograms (EEGs) intending for the same label.


During processing, the timespan for each label’s decoding is less than 100 ms (refer to Results); thus adjacent EEGs entering the algorithm should have an overlap. Given that there exists *x*% decoded labels consistent with the current *n*th, the timing diagram for instruction generation is provided in Figure 7. The theoretical timespan counting from the EEGs collection to the instruction generation is as follows:

(7) Ts=100+n×ts⁢(ms)

where Ts is the timespan of one instruction, ts is the timespan of one decoded label, and *n* is set to be 20 in this study comprehensively considering both the robustness and the rapidity.

## Results

### Effective connectivity of slight-facial expressions-electroencephalograms

To assess the particularity of the brain connectivity under sFEs, effective connectivity with the resting-state-EEGs was first computed as a standard. Such a resting-state threshold was calculated as the 99th percentile gOPDC to eliminate outliers and form a connectivity baseline. By comparing the threshold, only higher gOPDCs were retained to emphasize the stronger connectives. To reduce the computation, EEGs were down-sampled to 512 Hz.

**FIGURE 8:**
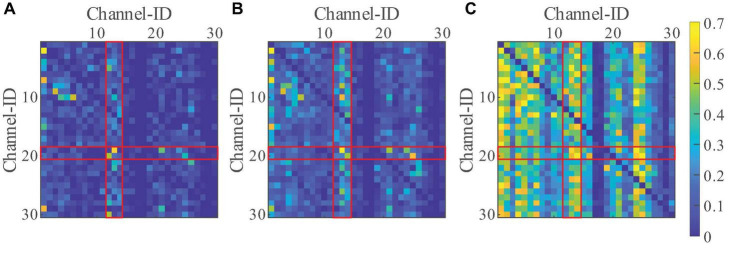
The normalized slight-facial expression (sFEs)-averaged generalized orthogonalized partial directed coherence (gOPDC) over normal threshold of **(A)** low frequency, **(B)** medium frequency, and **(C)** high frequency, where the value between the same channels were assigned as “NaN,” and the Channel ID.1 to ID.30 were listed as: Fp1, Fp2, Fz, F3, F4, F7, F8, FC1, FC2, FC5, FC6, Cz, C3, C4, T3, T4, CP1, CP2, CP5, CP6, Pz, P3, P4, T5, T6, PO3, PO4, Oz, O1, and O2. The block in *i*th row and *j*th column represents the gOPDC from Channel ID.*j* to Channel ID.*i*.

Filtering algorithm based on NA-MEMD and SampEn was used to remove artifacts, as detailed in our previous study (Li R. et al., 2018). Focusing on the typical frequency range of EEGs, gOPDCs were averaged into three representative bands as low frequency (4 Hz, 12 Hz), medium frequency (12 Hz, 30 Hz), and high frequency (30 Hz, 50 Hz) (Zavaglia et al., 2006). Figure 8 illustrates the normalized sFE-averaged connectivity of three EEG bands. Among the three bands, high frequency shows the strongest connectivity. Among all channels, electrodes arranged on the motor cortex show relatively more information exchange than others (where ID.12-14 Cz and C3 and C4 show more information outflow and ID.19-20 CP5 and CP6 show more information inflow).

**FIGURE 9:**
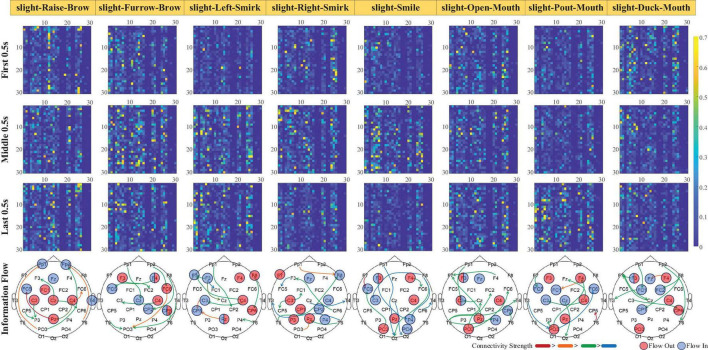
The dynamic change of normalized subjects-averaged generalized orthogonalized partial directed coherence (gOPDC) during slight-facial expression (sFE), and the directed information flow with 90% percentile connectivity and channels with more interaction. The list of Channel ID.1 to ID.30 is the same as in Figure 8, and the blocks in *i*th row and *j*th column represent the gOPDC from Channel ID.*j* to Channel ID.*i*.

Focusing on the dynamic process of sFEs, gOPDCs during the first 0.5 s (from 7.00 s to 7.50 s in each trial), the middle 0.5 s (from 8.75 s to 9.25 s) and the last 0.5 s (from 10.50 s to 11.00 s) were assessed in detail to reveal the connectivity changes from the very beginning to the end. Directed information flows with gOPDCs higher than other 90th percentile were marked in Figure 9, along with the channels performing more information interactions.

Among all 30 channels, those present inside and surrounding the motor cortex show more involvement and interactions. Typically, in slight-Left-Smirk, more interactions flow out of the right area into the left area of the brains; while in slight-Right-Smirk, more interactions flow from the right to the left. The participation of the visual cortex may result from the UI prompt. Some small asymmetries between the channels on the left and the right regions may be caused by the majority of right-handedness.

### Component analysis of slight-facial expressions-electroencephalogram

To analyze the component of sFE-EEG and form the guidance on signal processing, different passbands were selected to clarify the variation of signal components along with the frequency of sFE-EEG.

**FIGURE 10:**
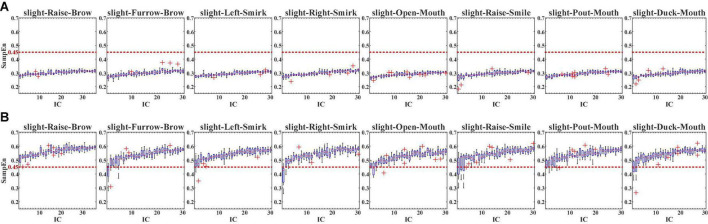
The SampEn of in-control (ICs) separated from slight-facial expression (sFEs)- electroencephalograms (EEGs) with **(A)** passband (5 Hz, 50 Hz) and **(B)** passband (5 Hz, 95 Hz). The red dash line represents the 0.45 threshold.

For the multi-channeled joint computing, FastICA was conducted first. With FastICA, the collected 30-channeled sFE-EEG were separated into 30 independent components (ICs). The selected non-quadratic non-linear function in FastICA is tanh, and the optimization function is Gauss. Figure 10 demonstrates the SampEn of each IC separated from sFE-EEGs.

In Figure 10A, with pass-band as (5 Hz, 50 Hz), it is clear that none SampEn of the 30 ICs among eight sFEs exceeds the 0.45 threshold, indicating the dominance of EEG information in each IC. While in Figure 10B, with the pass-band increased into (5 Hz, 95 Hz), the SampEn rises, with nearly all ICs exceeding the threshold, indicating the presence of EMG information. Such comparison demonstrated that, in the typical EEG bands lower than 50 Hz, the EEG components dominate the sFE-EEGs; while in the high-frequency bands above 50 Hz, more EMG components are engaged and influence the signal.

**FIGURE 11:**
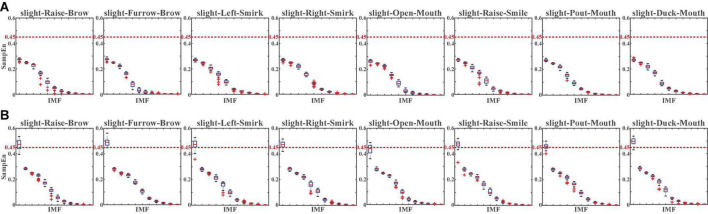
The SampEn of intrinsic mode functions (IMFs) decomposed from the slight-facial expression (sFEs)-electroencephalograms (EEGs) with **(A)** passband (5 Hz, 50 Hz) and **(B)** passband (5 Hz, 95 Hz). The red dash line represents the 0.45 threshold.

For a more specifically single-channel analysis, the NA-MEMD was applied to each channel to observe the SampEn of each intrinsic mode function (IMF). With NA-MEMD, the sFE-EEGs in each channel were decomposed into several IMFs, and each IMF whose SampEn exceeds 0.45 would be identified as the main carrier of EMG information. Figure 11 illustrates the SampEn of IMFs decomposed from the sFE-EEGs.

**FIGURE 12:**
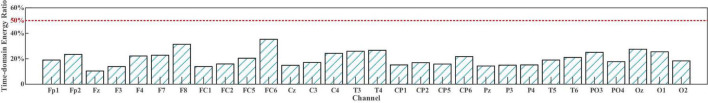
The decreased ratio of temporal energy in (5Hz, 95Hz) slight-facial expression (sFE)-electroencephalograms (EEG) after discarding the electromyogram (EMG) component.

Similarly, the comparison in Figure 11 demonstrates the same result as Figure 10. In passband lower than 50 Hz, none SampEn exceeded 0.45, indicating that EEG components dominate the sFE-EEGs; while in the high-frequency bands, the first IMF is the main carrier of EMG information, indicating that more EMG components are evolved. For passband (5 Hz, 95 Hz), by discarding those IMF whose SampEn exceeds the threshold and reconstructing the clean EEG, compared with the original sFE-EEG in (5 Hz, 95 Hz), the reduction ratios in temporal energy are shown in Figure 12.

After discarding the EMG component, compared to the original sFE-EEG in (5 Hz, 95 Hz), the average decline rate of temporal energy among all channels is 20.2% ± 5.7; in other words, nearly 80% of sFE-EEG’s original temporal energy is retained. The results above indicate that most of the energy of sFE-EEG comes from the EEG component; meanwhile, the EMG components mainly exist in a high frequency above 50 Hz.

### Asynchronous slight-facial expressions-paradigm interface

To determine the algorithm parameters and develop the sFE-paradigm software, data collected from offline experiments from six subjects were used in the offline assessment first. Then, 10 subjects participated in the online manipulation to evaluate the practical ability of such EEG-based control paradigm. During the offline assessment, the accuracy, precision, and time cost are given more consideration. While in the online evaluation, the controllability and the completion quality of tasks are more emphasized.

#### Offline datasets

**FIGURE 13:**
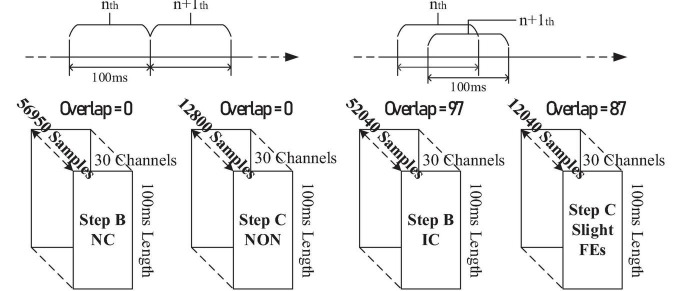
The illustration for datasets in offline assessment.

Data from the offline experiment were sliced into pieces and stacked. The imbalance for training datasets (in *Step B* and *Step C*) was solved by overlap, as illustrated in Figure 13.

Within *Step B*’s training, the NC dataset consists of EEGs from the resting-state, relaxing-state, eight facial-expressions with regular amplitude, and seven non-state-switching sFEs, while the IC dataset was only generated from state-switching sFE-EEGs. In *Step C*’s training, the dataset for “NON” was sliced from the resting-state EEG.

#### Offline assessment

##### Step A: Obvious non-slight-facial expressions-electroencephalograms exclusion

**FIGURE 14:**
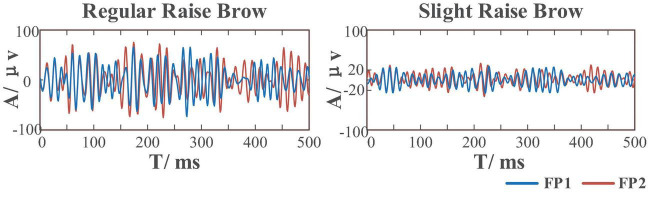
Comparison between electroencephalograms (EEGs) when performing regular-raise-brow vs. slight-raise-brow in (55 Hz, 95 Hz) of channel FP1 and FP2.

Compared to the former FE-BCI (Lu Z. F. et al., 2018), by limiting the range of facial muscle movement, this sFE-paradigm greatly reduces the proportion of EMG artifacts thus ensuring the possibility to distinguish between daily FE and sFE during control. Figure 14 shows the comparison between regular FE-EEGs vs. sFE-EEGs. The amplitude range of sFE-EEGs is much smaller than that under regular facial-expressions. To exclude non-sFE-EEGs, several different methods for threshold setting are compared in Table 4.

**TABLE 4:** Comparison among different threshold setting methods.

Method	Formula	Correctly acceptance rate (AR)	Correctly rejection rate (RR)	Timespan (Ts)	Score*
Maximum	$\max\left\{\left|x_{i}\right|\right\},\;\left(i=1,\ldots ,100\right)$	99.48%	64.67%	0.0055ms	39.11
Temporal energy	$\sum_{i=1}^{i=100}x_{i}^{2}$	99.45%	68.66%	0.0055ms	40.53
Amplitude range	$\max\left\{\left|x_{i}\right|\right\}-\max\left\{\left|x_{i}\right|\right\},\;\left(i=1,\ldots ,100\right)$	99.48%	64.80%	0.0055ms	39.15

*The score is calculated as 1/(1/AR + 1/RR + Ts).

Throughout the three methods, the temporal energy achieves the highest score. By setting the threshold as the temporal energy, the correctly acceptance rates (ARs) of different sFEs are listed in Table 5. ARs in Table 5 are nearly 99% and show no typical imbalances. The extremely short timespan, high AR, and high RR prove the feasibility of threshold comparison as the first segment of the algorithm.

**TABLE 5:** Correctly acceptance rate of eight slight-facial expression (sFEs) with threshold as temporal energy.

S	s-RB* (%)	s-FB* (%)	s-LS* (%)	s-RS* (%)	s-OM* (%)	s-S* (%)	s-PM* (%)	s-DM* (%)
S1	97.19	100	99.94	99.25	100	99.31	99.56	99.94
S2	98.38	98.88	99.75	100	99.94	99.06	99.94	99.25
S3	98.63	100	98.81	98.81	100	100	100	100
S4	98.06	99.94	98.88	99.75	100	99.06	99.94	100
S5	97.94	99.88	100	99.94	100	99.94	99.75	98.00
S6	97.75	100	99.00	99.13	99.88	100	99.94	100

*s-RB stands for slight-Raise-Brow; s-FB for slight-Furrow-Brow; s-LS for slight-Left-Smirk; s-RS for slight-Right-Smirk; s-OM for slight-Open-Mouth; s-S for slight-Smile; s-PM for slight-Pout-Mouth; s-DM for slight-Duck-Mouth.

##### Step B: Interface “ON” detection

To select one sFE as the most suitable sFE for state-switching, eight sFEs were first compared using SVMs with four different kernel functions, as listed in Table 6. In Table 6, the s-RB achieves the highest overall accuracy when it is set as the state-switching sFE. The detailed performances of SVM kernels are listed in Table 7, with the s-RB as the state-switching sFE. Additionally, the time cost of CSP feature engineering is 0.1328 ms in average.

**TABLE 6:** The Accuracy for eight different state-switching- slight-facial expression (sFEs).

sFE	SVM kernel			
	Linear polynomial (%)	Quadratic polynomial (%)	Scaled gaussian (%)	Medium gaussian (%)
s-RB	96.19	74.59	96.46	96.30
s-FB	80.90	50.36	84.07	83.28
s-LS	76.70	51.54	81.08	80.60
s-RS	80.23	52.85	83.33	82.75
s-OM	72.84	53.28	81.66	81.41
s-S	71.49	50.23	78.37	77.27
s-PM	68.95	49.55	75.32	74.68
s-DM	66.95	49.14	77.36	76.76

**TABLE 7:** The detailed performance of different SVM kernels*

SVM kernel	Accuracy (%)	Precision(%)	Recall (%)	F1 score	Prediction speed	Training duration
Linear polynomial	96.19	94.58	98.02	48.14	490000obs/s	>15min
Quadratic polynomial	74.59	75.62	73.23	37.20	96000obs/s	>15min
Scaled gaussian	96.46	95.29	97.78	48.26	130000obs/s	<3min
Medium gaussian	96.30	94.82	97.98	48.19	170000obs/s	<2min

*Evaluation with Win10, i5-6500, 3.20GHz, without GPU accelerated.

Among four SVM kernels, the linear polynomial, the scaled Gaussian, and the medium Gaussian achieve higher F1 scores (Derczynski, 2016). Comprehensively considering the F1 score, prediction speed, and training duration, the scaled Gaussian performs the best. The adaptability of scaled Gaussian kernel to each subject is listed in Table 8. The accuracy, precision, and recall in Table 8 show a relatively small standard deviation (SD) among subjects. Based on all the assessments above, the CSP combining with scaled-Gaussian-SVM proves its best applicability, which maintains a stable performance without obvious individual differences.

**TABLE 8:** The accuracy, precision and recall with scaled gaussian.

S	Accuracy (%)	Precision (%)	Recall (%)
S1	97.79	96.14	99.57
S2	96.47	95.22	97.85
S3	96.62	97.09	96.13
S4	94.60	92.50	97.06
S5	97.11	95.65	98.70
S6	96.18	95.13	97.34
Mean	96.46	95.29	97.78
Std	±1.07	±1.54	±1.22

##### Step C: Slight-facial expressions-electroencephalograms real-time decoding

As a multi-classification with nine targets based on deep-learning, several mature structures are assessed to verify their applicability first, such as VGG16 (Simonyan and Zisserman, 2014), DenseNet121 (Huang et al., 2017), and ResNet50 (He et al., 2016). To match with this specified problem, all network applications were imported without pretrained weights and slightly modified, as shown in Table 9. Compared with the sFE-Net, their accuracies under 10-fold validation are listed in Table 10. Through comparison, DenseNet121 and ResNet50 both achieve not only higher training accuracy, but also face serious overfitting problems (even would suddenly drop to a 40% validation accuracy in the late-training-process). Compared with three mature network applications, the sFE-Net, although not the highest training accuracy, shows a more balanced performance between training and validation.

**TABLE 9:** Structure for network applications.

Layer (type)	Method	Parameter	Value
Network applications	–	Pretrained weights	None
		Top layer	Not include
Dense	–	Units	256
		Activation	ReLU
Dropout	–	–	0.5
Dense	One hot	Units	9
		Activation	Softmax
Loss function	Categorical cross entropy	–	–
Optimizer	RMSprop	Learning rate	0.001
Training acceleration	Batch	Batch size	128

**TABLE 10:** Performance comparison among deep-learning structures.

Deep learning network	Training accuracy (%)	Validation accuracy (%)
VGG16	11.03	11.34
DenseNet121	98.91	88.94
ResNet50	98.52	87.27
sFE-Net	92.68	89.48

**FIGURE 15:**
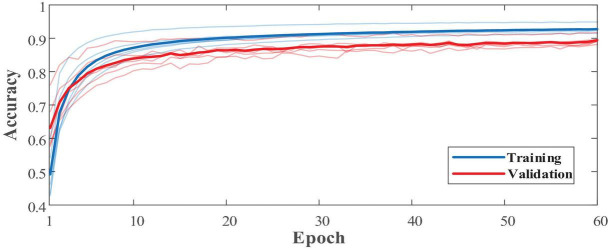
The learning process of slight-facial expression (sFE)-Net.

In addition to the network structure, extra feature engineering steps were combined to assess its effectiveness (Table 11). Since the sFE-Net model is not designed to accept 4-dimensional input tensors, the classifier combined with the filter bank common spatial pattern (FB-CSP) (Ang et al., 2008) feature engineering was selected to be the DenseNet121. In Table 11, the sFE-Net without any hand-made feature engineering achieves the best performance. The iteration of sFE-Net is shown in Figure 15. The detailed accuracy of sFE-Net under 10-fold validation and its prediction speed are listed in Table 12.

**TABLE 11:** Performance comparison for extra feature engineering.

Method	Feature engineering	Classifier	Training accuracy (%)	Validation accuracy (%)
A	CSP (One vs. Rest)	SVM (Fine Gaussian)	74.24	71.45
B	FB-CSP (Three frequency bands)	DenseNet121	89.87	36.63
C	–	sFE-Net	92.68	89.48

**TABLE 12:** Offline accuracy of slight-facial expression (sFE)-Net with 10-fold validation*.

S	Training accuracy (%)	Validation accuracy (%)	Prediction speed
S1	92.58	89.59	256000obs/s
S2	92.45	89.06	256000obs/s
S3	91.21	88.00	257000obs/s
S4	92.25	89.34	254000obs/s
S5	94.89	91.69	254000obs/s
S6	92.69	89.17	256000obs/s
Mean	92.68	89.48	–
SD	±1.21	±1.21	–

*Evaluation with Win10, i5-6500, 3.20GHz, accelerated with GTX 960.

**FIGURE 16:**
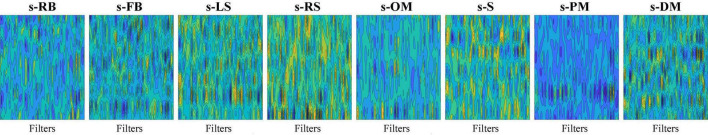
The contoured feature maps extracted by the convolutional neural network (CNN) layer in slight-facial expression (sFE)-Net.

As demonstrated in Table 12, the sFE-Net achieves a stable performance varying among subjects with a ±1.21% Std; Meanwhile it maintains a precise accuracy and relatively small overfitting (with the validation accuracy as 89.48% and the training accuracy as 92.68%). The average time cost for one prediction is nearly 0.235 ms. Figure 16 demonstrates the contoured feature maps extracted by the CNN layer in sFE-Net. The confusion matrix among eight sFEs (seven control targets and one state-switching target) is listed in Table 13.

**TABLE 13:** Confusion matrix among eight slight-facial expression (sFEs).

True label	Predicted label								
	s-RB (%)	s-FB (%)	s-LS (%)	s-RS (%)	s-OM (%)	s-S (%)	s-PM (%)	s-DM (%)	NON (%)
s-RB	99.15	0.07	0.00	0.05	0.05	0.01	0.01	0.03	0.63
s-FB	0.08	94.91	0.63	0.48	0.44	0.27	0.21	0.05	2.93
s-LS	0.03	1.02	92.17	0.51	0.39	1.12	0.33	0.21	4.23
s-RS	0.05	0.22	0.78	94.05	0.20	1.38	0.20	0.27	2.85
s-OM	0.10	0.39	0.25	0.18	93.23	0.22	0.82	0.66	4.14
s-S	0.07	0.13	0.99	0.83	0.27	93.20	0.96	0.92	2.63
s-PM	0.09	0.23	0.43	0.16	1.07	1.24	91.61	0.90	4.27
s-DM	0.05	0.13	0.48	0.36	1.07	1.15	1.05	90.20	5.51

Among the eight sFEs, the s-RB (corresponding to state-switching) achieves the highest prediction accuracy of 99.15%, While the lowest of 90.20%is achieved by s–S. The averaged prediction accuracy among such eight sFEs is 93.56%. In each true label, the confusion with “NON” is especially higher than the others, which decreases the averaged classification accuracy. The high misrecognition rate between the true label and “NON” must be caused by a small change between the sFEs and the resting-state, which results in similar signal ingredients. Considering the advantage to retain the “NON” label as a hold-on command, which provides the system a rest even after entering the IC state, such “NON” label cannot be deleted and is designed to be remained. The stability of the final instruction is enhanced by *Step D* to improve accuracy.

##### Step D: Validity judgment

As has been assessed in detail above, the timespan (with ample margin) of each step is summarized in Table 14. The theoretical timespans for different instructions are all less than 200 ms, which meet the specified requirement for real-time control.

**TABLE 14:** Timespan (with ample margin) of each step.

Step	Timespan (ms)
*Step A*: Obvious non-sFE-EEGs exclusion	≈ 0.01
*Step B*: Interface ‘ON’ detection	≈ 0.15
*Step C:* sFE-EEGs real-time decoding	≈ 0.24

According to the theoretical time cost for generating one instruction in Eq. (7), the timespan for different instruction is listed in Table 15.

**TABLE 15:** Theoretical timespan for instruction generation.

Instruction	Theoretical timespan (ms)
Asynchronous interface “ON”	≈103.2
Asynchronous interface “OFF”	≈105.0
sFE-paradigm based control instruction	≈105.0

#### Online evaluation

Based on the offline assessment, an sFE-paradigm software has been developed to facilitate the easy application of the online external device control. By using this software, the subject-dependent classifiers and parameters were automatically computed, and then the online sFE-paradigm controlling task with AUBO-i5 robotic arm system and the 2-DoFs prosthetic hand were conducted. Within the stepping control strategy adopted by these two peripherals, the stepping distances are not elaborately designed for tasks. Due to the visual perspective, stepping distance, and some unavoidable random errors, these tasks can hardly be completed with perfect zero deviation, even by using the referred control method (i.e., FlexPendant and Joystick). In the following online evaluation, the practicality and the controllability of the sFE-paradigm are more concerned (e.g., the floating range of the indicator) than the precision. Compared between these two tasks, *Task one* requires higher stability, while *Task two* emphasized more on agility and real-time ability.

##### Task one: Object-moving with a robotic arm

**FIGURE 17:**
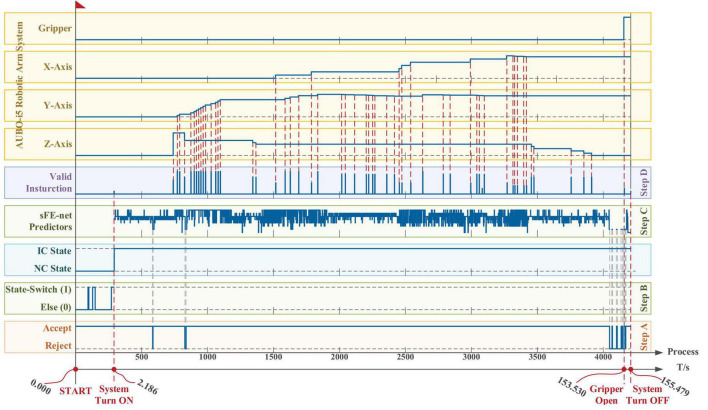
The time log of a successful online manipulation in *Task one* performed by subject S7. From bottom to top, the first axis is the timeline, and the second axis is a counter which represents the processing of nth 100 ms EEGs. The five boxes in the lower area illustrate the algorithm results along the timeline step-by-step, and the four boxes in the upper area illustrate the systematic movement of the AUBO-i5 robotic arm. The *x*, *y*, and *z* axes for the robotic arm system of AUBO-i5 are consistent with Figure 4A, and the timelines for all axes and all boxes remain aligned.

To manipulate the AUBO-i5 robotic arm system, all eight sFEs were enabled. Considering the different important levels of instructions (e.g., a trial would be judged as a failure once the electric gripper is mistakenly opened halfway), the sFE with the highest recognition accuracy (except for the s-RB) is assigned to the most vital instruction. The correspondence between the sFEs and the system instruction is listed in Table 16, where s-RB is responsible for the state-switching as mentioned before. Table 16 also lists the consistency checking criteria. Figure 17 demonstrates the time log of a successful online manipulation for *Task one* performed by subject S7.

**TABLE 16:** The operational correspondence, along with the consistency checking criteria *x*% in *Task one.*

Item	s-RB	s-FB	s-LS	s-RS	s-OM	s-S	s-PM	s-DM
Instruction	–	Open	Left	Right	Up	Down	Backward	Forward
Criteria	0.95	0.95	0.45	0.45	0.45	0.95	0.55	0.80

**FIGURE 18:**
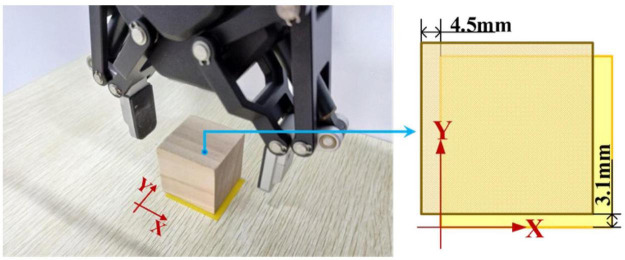
The illustration for placement deviation in *Task one*.

**FIGURE 19:**
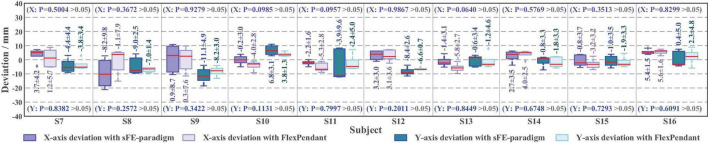
The boxplot of placement deviation in *Task one* with slight-facial expression (sFE)-paradigm and FlexPendant, where the short red line indicates the median, and the number indicates the mean.

According to Figure 17, first the timestamp 0.000 s begins at the ‘Start’ button being pressed by S7-self; second, at 2.186 s S7 switched on the sFE-paradigm successfully; third, after careful movements, the S7 opened the electrical gripper to put down the wooden block at 153.530 s; finally, the S7 switched off the sFE-paradigm and finished the task at 155.479 s. During the operation, unrelated EEGs were successfully rejected by sFE-paradigm system. The final deviation of the wooden block in the trial listed in Figure 17 is –4.5 mm on *x*-axis and +3.1 mm on *y*-axis (Figure 18). The placement deviation with the sFE-paradigm is shown in Figure 19, along with the deviation by FlexPendant.

From bottom to top, the first axis is the timeline, and the second axis is a counter which represents the processing of *n*th 100 ms EEGs. The five boxes in the lower area illustrate the algorithm results along the timeline step-by-step, and the four boxes in the upper area illustrate the systematic movement of the AUBO-i5 robotic arm. The *x*, *y*, and *z* axes for the robotic arm system of AUBO-i5 are consistent with Figure 4A, and the timelines for all axes and all boxes remain aligned.

**FIGURE 20:**
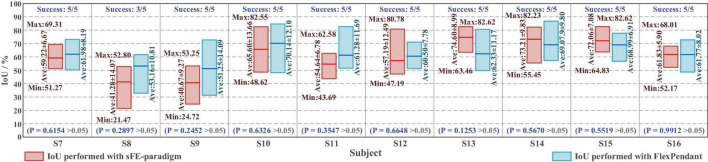
The intersection over union (IoU) of placement deviation in *Task one* with slight-facial expression (sFE)-paradigm and FlexPendant, where the short red line indicates the average.

For a reliable control method, under one’s visual guidance, the deviation shall cause certain random volatility within a small range. Instead of perfect zero deviation, the variation range of deviation under sFE-paradigm is of more concerned. Among all online subjects (S7-S16), the averaged variation range (AVR) of sFE-paradigm’s deviation is 11.14 ± 6.13 mm along *x*-axis and 9.78 ± 3.83 mm along *y*-axis, and 10.46 ± 5.15 mm in total (while the AVR of FlexPendant is 7.75 ± 7.67 mm in total and shows no significant difference with *P* = 0.2089 > 0.05). As in Figure 19, for most subjects, compared with FlexPendant, deviation by sFE-paradigm achieves a comparable range of variation, a similar averaged value, and a close Std, which indicates an approximative level of controllability as FlexPendant. But for few subjects, on one certain axis (i.e., the *x*-axis of S11) where the median of sFE-paradigm’s boxplot is marked far from the average, it indicates that there exists uncontrollability during the position adjustment along that axis by using the sFE-paradigm. Overall, the maximum deviation with sFE-paradigm can largely be limited below 20 mm (S8), from which the minimum deviation can sometimes be ±0.1 mm (S10, S11), and none of the subject deviations shows significant difference compared between sFE-paradigm and FlexPedant. The intersection over union (IoU) between the target location B and the block placement in *Task one* is demonstrated in Figure 20.

Among all the 50 trials, 96% of trials succeed, only 2 trials by S8 failed halfway. The averaged IoU of sFE-paradigm is 60.03 ± 11.53%, while 62.05 ± 6.01% of FlexPendant, shows no significant difference with *P* = 0.6521 > 0.05. As in Figure 20, the minimum IoU with sFE-paradigm among all is 21.47% (S8) while the maximum is 82.62% (S13 and S15). Compared with FlexPendant, the statistical test indicates that the IoU of sFE-paradigm shows similar performance overall. But for several subjects (e.g., S8, S9, S11, and S14), the IoU of FlexPendant performed slightly better and scored higher than sFE-paradigm, which indicates that during an actual application process, the operation under sFE-paradigm is more complicated.

**FIGURE 21:**
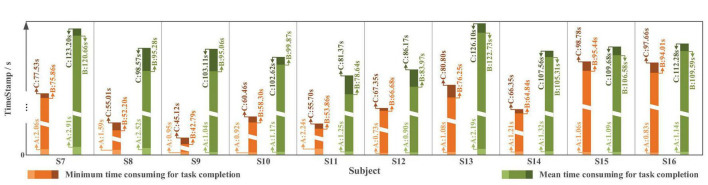
The time cost in *Task one* with slight-facial expression (sFE)-paradigm, where the timestamp A corresponds to “System Turn ON,” the timestamp B corresponds to “Gripper Open” and the timestamp C corresponds to “System Turn OFF” (as demonstrated in Figure 17).

In terms of time cost (Figure 21), affected by different levels of tension and proficiency, the completion time varies from less than 60 s to approximately 150 s. The average completion time for *Task one* is 105.07 ± 13.50 s; in which, for “System Turn ON” stage is 1.55 ± 0.67 s, for block moving and placing stage is 100.19 ± 13.17 s and for “System Turn OFF” stage is 3.27 ± 1.64 s. The time-consuming of each stage starts from the end of the previous state (or valid instruction) to the beginning of the next state (or valid instruction).

##### Task two: Water-pouring with a prosthetic hand

With a 2-DoFs prosthetic hand, only four sFEs that achieved higher decoding accuracy were enabled. Apart from these four sFEs, other valid instructions generated from the rest sFEs would not be transmitted. Slightly differing from *Task one*, *Task two* requires a higher real-time response, because a tiny change in wrist angle will pour several milliliters or even dozens of milliliters of water. Table 17 shows the correspondence between the sFEs and the instruction (left-handed prosthesis), and the consistency checking criteria. Figure 20 demonstrates the time log of a successful online process in *Task two*.

**TABLE 17:** The operational correspondence and the consistency checking criteria in *Task two*.

Item	s-RB	s-FB	s-LS	s-RS	s-OM	s-S	s-PM	s-DM
Instruction	–	Open	Extorsion	Intorsion	–	Close	–	–
Criteria	0.95	0.95	0.35	0.40	1	0.95	1	1

**FIGURE 22:**
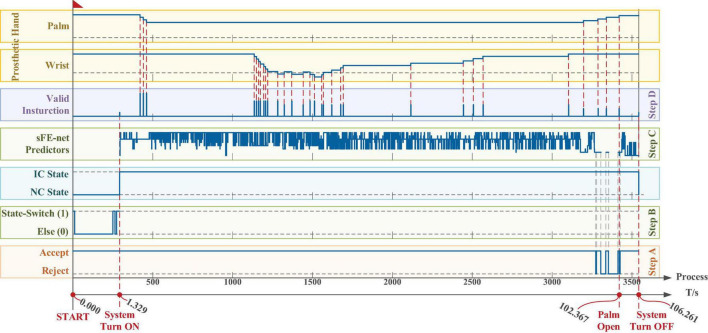
The time log of a successful online manipulation in *Task two*. From bottom to top, the first axis is the timeline, and the second axis is a counter which represents the processing for *n*th 100 ms electroencephalograms (EEGs). The five boxes in the lower area illustrate the algorithm results along timeline step-by-step, and the two boxes in the upper area illustrate the 2-DoFs prosthetic hand movement. The timelines for all axes and all boxes remain aligned.

In Figure 22, subject switched on the sFE-paradigm at 1.329 s, then completed the water-pouring task at 102.367 s, and finally switched off the interface at 106.261 s; The final deviation of water volume in the trial listed in Figure 22 is less than 1 ml.

**FIGURE 23:**
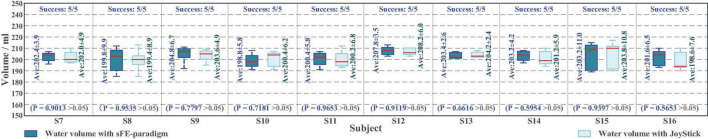
The boxplot of water volume in online experiment *Task two* with slight-facial expression (sFE)-paradigm and JoyStick, where the short red line indicates the median, and the number indicates the mean.

**FIGURE 24:**
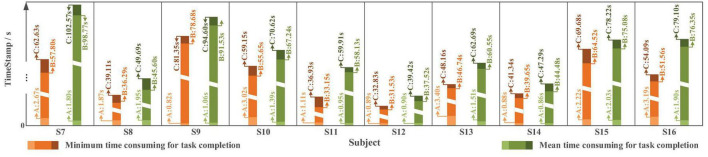
The time spent in the online experiment for *Task two* with the slight-facial expression (sFE)-paradigm, where the timestamp A corresponds to ‘System Turn ON’, the timestamp B corresponds to ‘Palm Open,’ and the timestamp C corresponds to ‘System Turn OFF’ (as demonstrated in Figure 22).

Same as *Task one*, the deviation affected by the visual perspective and the step distance can hardly be zero even by Joystick. The water volume in *Task two* is demonstrated in Figure 23. Among all 50 trials of *Task two*, 100% trials successfully finished. The averaged water volume by sFE-paradigm is 202.5 ± 2.5 ml, while 202.2 ± 2.7 ml similarly by Joystick, between which shows no significant difference with *P* = 0.7931 > 0.05. Compared with Joystick, with such simplified 4 instructions, the sFE-paradigm achieved almost the same performance as Joystick in water pouring task. The average completion time of *Task two* is shown in Figure 24.

The time spent varies from approximately 60 to 150 s. The average completion time for online *Task two* is 68.41 ± 19.52 s, while the averaged time spent for “System Turn ON” stage is 1.43 ± 0.45 s, for water pouring stage is 64.09 ± 19.06 s, and for “System Turn OFF” stage is 2.88 ± 0.74 s.

## Discussion

### Potential influencers of online performance

To explore the practicality of the developed sFE-paradigm, attention was paid to its online application procedure. In the online study, there were the following three factors that had been noticed which would affect the performance, and for which our research group planned to conduct a more in-depth study.

#### Long-term wearing of electroencephalogram cap

Tightened by the EEG cap fabric, the subjects experienced varying degrees of itching, leading to subconscious scratching, which became more frequent over time. Meanwhile, in the manipulation, under a relatively concentrated state, several subjects sweated beneath the EEG cap. In the whole online experiment, the subjects might eat (or even have dinner) with the EEG cap worn, and some subjects would move the strap fixed to their chin to facilitate chewing.

Reasons above caused the shift of EEG electrodes which enlarges with the increase of wearing time. Such a shift resulted in the difference between the EEG detected in online and collected during acquisition. In terms of impedance, as the wear duration lengthens, the impedance was reduced to lower than 1 kΩ; meanwhile, the conductive gel got solidified and dried up. Noticing these, with the proceeding of the online procedure, we have gradually reduced the amount of training data gathering to compress the duration.

#### Mental state

Being more difficult than commercial control forms (i.e., FlexPendant and Joystick), the subjects were relatively more concentrated in the process of sFE-paradigm-based control tasks, resulting in varying nervousness depending on personal psychology. In operation, subjects with higher levels of tension were found to show greater degradation from the “debug” mode (sFE- paradigm without peripherals connected) to the actual sFE-paradigm-based electromechanics control; On the contrary, the performance of tranquil subjects remained consistent. In addition, by lessening the control instructions, *Task two* shows a decrease in difficulty, the relief of nervousness, and the improvement of proficiency. Thus, even *Task two* requires more operation agility, and the overall performance is more excellent than *Task one*.

In the whole online study, we have observed that the mental state of subjects has a great impact on the completion quality of control tasks, inspired by which we have also carried out a study on the compensation method of manipulation quality affected by mental state.

#### Physical movements

In the online procedure, the subjects were asked to sit comfortably, but to avoid extra-large physical movements. For most subjects, body movements were found that would reduce the stability of sFE-paradigm during applications. But for few subjects, such as subjects S11 and S14 who were actively asked to stand up, pace, and softly communicate in *Task one*, physical movements did not show much influence on the stability of the sFE- paradigm. The performance of these trials by subjects S11 and S14 were listed in the result of this study. Encouraged by these two subjects, we believe that the proposed sFE-paradigm is promising of realistic applicability, thereby emphasizing a future study on the stability under physical movements of sFE-paradigm.

### The balance between robustness and real-time

In the online tasks, the timer started at the “Start” button (on the GUI) being pressed by the subjects and ended at the sFE-paradigm being switched off by the subjects. All participants were not well-skilled BCI users and had no prior experience with the sFE-paradigm. Each time-spent as provided in Figures 21 and 24 can be regarded as the time-consuming of one stage (instead of merely the signal procedure for 20 continuous decoded targets). For the turn-on stage, since the GUI was interacted with the mouse, from the timestamp 0.00 s to the successful switch-on of the sFE-paradigm, it contained the time of releasing the mouse, self-mental adjustment, and activity, and slightly raising-brow to start the paradigm. Similarly, for the turn-off stage, starting from sending the last equipment operation instruction and ending up at successfully switching off the system, it contained the time of completing the equipment’s movement according to the instruction, self-mental adjustment, and slightly raising-brow to switch off the paradigm.

In this study, based on the numerical criteria of real-time ability, the time length for EEG inputs was selected; meanwhile, to reduce the misoperation, the validity judgment (for generating one valid instruction) depended on 20 continuous decoded targets was designed, of which its theoretical time-spent met the real-time requirements. However, in the online manipulation, when interacting with the electrical equipment in real work tasks, being affected by personal mental states and levels of tension, the complexity of EEGs increased, and the stability decreased. By taking the system robustness as the priority, the consistency checking criteria were set at a risk-free level. Thus, not each continuous 20 decoded targets can meet the validity judgment and generate one instruction, hence resulting in different levels of real-time ability which do not always reach the theoretical ideal.

By presenting the whole operation procedure of the sFE-paradigm-based real task operation, in this study, the online performance emphasis was placed on the controllability and agility (whether it reached the same level as other commercial control methods). In further studies, to optimize the paradigm capability, a reinforcement learning would be adopted to adaptively adjust the validity judgment policy according to the current working conditions, to find a better scheme in balancing the real-time ability and robustness.

## Conclusion

In this study, a novel asynchronous artifact-enhanced EEG-based control paradigm assisted by slight-facial expressions with eight valid control instructions was proposed and implemented. Through the insight of brain connectivity analysis, the high participation of the motor cortex under sFE-paradigm was revealed, which was conformed to the contralateral control fact and demonstrated the domination of motor cortex. The component analysis with sFE-EEG indicated the dominance of EEG components in sFE-EEG. The sFE-paradigm proved its feasibility and practicality through various online electromechanical manipulation tasks, focusing especially on stability and agility. Both offline and online results demonstrated the capability of sFE-paradigm. In the offline procedure, it achieved accuracies of 96.46% ± 1.07 for interface switching and 92.68% ± 1.21 for real-time control, with a 105.5 ms theoretical timespan for sFE-paradigm -based instruction generation. In online procedure, the sFE-paradigm performed 60.03 ± 11.53% averaged IoU in *Task one* and 202.5 ± 7.0 ml averaged water volume in *Task two*. Results suggested that the sFE-paradigm showed the similar level of controllability and agility as FlexPendant (*P* = 0.6521 > 0.05) and Joystick (*P* = 0.7931 > 0.05). During the study, more subtle factors that may affect online performance have been noticed by us, on which we will carry out more in-depth research. We sincerely look forward to the realization of neuro-based control in real-world applications in the future.

## Data availability statement

The raw data supporting the conclusions of this article will be made available by the authors, without undue reservation.

## Ethics statement

This studies involving human participants were reviewed and approved by the institutional review board of Xi’an Jiaotong University (No. 20211452), and all experiments were conducted in accordance with the declaration of Helsinki. The patients/participants provided their written informed consent to participate in this study. Written informed consent was obtained from the individual(s) for the publication of any potentially identifiable images or data included in this article.

## Author contributions

ZL: proposed and conducted the research and wrote the manuscript. XZ: supervised this study and revised the manuscript. HL and TZ: organized and carried out the experiments. LG and QT: revised the manuscript. All authors contributed to the article and approved the submitted version.
